# Development of a risk model based on autophagy-related genes to predict survival and immunotherapy response in ovarian cancer

**DOI:** 10.1186/s41065-023-00263-2

**Published:** 2023-02-01

**Authors:** Yuwei Chen, Zhibo Deng, Yang Sun

**Affiliations:** 1 Department of Gynecology, Clinical Oncology School of Fujian Medical University, Fujian Cancer Hospital, Fuzhou, China; 2 Department of Orthopedics, Shengli Clinical Medical College of Fujian Medical University, Fujian Provincial Hospital, Fuzhou, China

**Keywords:** Autophagy, Ovarian cancer, Tumor microenvironment, Drug sensitivity, Immunotherapy

## Abstract

**Background:**

Autophagy is a highly conserved cellular proteolytic process that can interact with innate immune signaling pathways to affect the growth of tumor cells. However, the regulatory mechanism of autophagy in the tumor microenvironment, drug sensitivity, and immunotherapy is still unclear.

**Methods:**

Based on the prognostic autophagy-related genes, we used the unsupervised clustering method to divide 866 ovarian cancer samples into two regulatory patterns. According to the phenotypic regulation pattern formed by the differential gene between the two regulation patterns, a risk model was constructed to quantify patients with ovarian cancer. Then, we systematically analyzed the relationship between the risk model and immune cell infiltration, immunotherapeutic response, and drug sensitivity.

**Results:**

Based on autophagy-related genes, we found two autophagy regulation patterns, and confirmed that there were differences in prognosis and immune cell infiltration between them. Subsequently, we constructed a risk model, which was divided into a high-risk group and a low-risk group. We found that the high-risk group had a worse prognosis, and the main infiltrating immune cells were adaptive immune cells, such as Th2 cells, Tgd cells, eosinophils cells, and lymph vessels cells. The low-risk group had a better prognosis, and the most infiltrated immune cells were innate immune cells, such as aDC cells, NK CD56dim cells, and NK CD56bright cells. Furthermore, we found that the risk model could predict chemosensitivity and immunotherapy response, suggesting that the risk model may help to formulate personalized treatment plans for patients.

**Conclusions:**

Our study comprehensively analyzed the prognostic potential of autophagy-related risk models in ovarian cancer and determined their clinical guiding role in targeted therapy and immunotherapy.

**Supplementary Information:**

The online version contains supplementary material available at 10.1186/s41065-023-00263-2.

## Introduction

Ovarian cancer is one of the most common malignant tumors of female reproductive organs. The prognosis is the worst among three common gynecological tumors, with incidence rate and mortality increasing year by year [1]. Because the ovary is deep in the pelvic cavity, ovarian cancer had no clinical symptoms in the early stage, and there was a lack of effective early diagnostic markers, most patients were diagnosed with advanced disease. According to cancer statistics in 2020, about 21,750 patients in the United States were diagnosed with ovarian cancer and 13,940 ovarian cancer-related deaths [2]. Although the combination of cytoreductive surgery and neoadjuvant chemotherapy increases the survival time of patients with ovarian cancer [3], the overall survival rate of patients with ovarian cancer is still at a low level [4]. The poor prognosis of ovarian cancer was caused by many factors, including advanced diagnosis, drug resistance to chemotherapy, and a high recurrence rate after treatment [5]. At present, the prognosis of patients with ovarian cancer is mainly based on pathological stage and differentiation. However, traditional methods could not accurately judge the prognosis of patients. Therefore, it’s very necessary to build a stable, accurate, and efficient prediction model to help doctors improve patients' treatment plans and prolong patients' survival time. In recent years, great efforts have been made to explore genomic mutations, abnormal transcriptome changes, and biological targets for disease diagnosis and treatment [6]. Among them, the important role of autophagy in tumor occurrence, metastasis, targeted therapy, and drug resistance has gradually attracted people's attention.

Autophagy is an important evolutionary process for eukaryotes to regulate intracellular substances. In this process, damaged proteins or organelles are wrapped in double-membrane structured gold-phagocytic vesicles, transported to lysosomes (animals), or degraded and recycled in vacuoles (yeasts and plants) [7]. Autophagy is essential for maintaining homeostasis. Therefore, the abnormality of autophagy regulation is related to a variety of diseases, such as tumors, neurodegenerative diseases, and cardiovascular diseases [8]. Autophagy is like a double-edged sword. It can promote or inhibit the progress of cancer according to the changes in the surrounding environment. Tumor type, stage, and treatment may affect the regulation of autophagy in a tumor. Generally speaking, autophagy can inhibit the occurrence and development of cancer by removing mutated genes, damaged proteins, and heterotypic cells in the body. However, in previous studies, the increase in autophagy level can usually recycle intracellular macromolecules and organelles to promote the special needs of tumor growth [7]. Many studies have explored the role of some autophagy-related genes in the occurrence and development of ovarian cancer [9, 10]. However, autophagy is a complex regulatory mechanism of the body, involving hundreds of genes. Previous studies only discussed the regulatory role of autophagy in ovarian cancer by changing the expression level of a single autophagy-related gene, which is not comprehensive enough. Constructing a model integrating multiple autophagy-related genes may improve the accuracy of prognosis prediction.

More and more studies show that the occurrence and development of the tumor are not only related to genetic factors but also inseparable from the control of the immune microenvironment [11]. Immunotherapy based on immune checkpoint inhibitor (ICB) has been applied to the treatment of cancer and achieved good clinical results. However, most patients have a low response to immunotherapy [12]. To make better application of immunotherapy, we urgently need to analyze the immune microenvironment and understand the reasons for patients' low response to ICB. Both autophagy and innate immune response can detect changes such as cell injury or infection to ensure homeostasis. Recent studies have shown that in tumor cells, the autophagy pathway is intertwined with pattern recognition receptor (PRR), inflammation, and cell death pathway, which can change the immunogenicity and antitumor immune response of TME, to enhance tumor clearance [13]. Therefore, analyzing the interaction mechanism between autophagy and immune response in the context of tumorigenesis may provide a new therapeutic target for enhancing antitumor immunity and immunotherapy.

In this study, we integrated the gene expression profiles of seven GSE ovarian cancer cohorts from the GEO database [14] and evaluated the patterns of autophagy regulation. We found that the autophagy regulation pattern was not only related to the infiltration of multiple immune cell types but also related to the prognosis of patients. Then, we analyzed the differentially expressed genes (DEGs) between different autophagy regulation patterns and constructed a risk score model to quantify the role of autophagy in a single ovarian cancer patient. Finally, we analyzed the prognostic role of the risk model in patients and evaluated its therapeutic role in immunotherapy and chemotherapy.

## Methods

### Data collection and processing of ovarian cancer samples

The clinical data and gene expression information of ovarian cancer samples were obtained from GEO (https://www.ncbi.nlm.nih.gov/geo/) and TCGA(https://portal.gdc.cancer.gov/) databases [15]. In the GEO database, we integrated seven eligible OV cohorts (GSE9891, GSE18520, GSE19829, GSE20565, GSE26193, GSE30161, GSE26712), and then used the TCGA-OV cohort for follow-up validation. The "combat" algorithm of the SVA package [16] was used to correct the batch effect caused by the system. Data analysis and mapping were completed by R and R-dependent packages.

We collected the data of the IMvigor 210 cohort [12] and GSE78220 cohort [17] for immunotherapy analysis. The expression data and clinical treatment information of the IMvigor210 cohort were downloaded from http://research-pub.Gene.com/imvigor210corebiologies, and the data were normalized by the DEseq2 R package. The data of the GSE78220 cohort were obtained from the GEO database, and the original data was standardized by the limma package.

### Unsupervised cluster analysis of autophagy-related genes

A total of 232 autophagy-related genes were obtained from the Human Autophagy Database (HADb, http://www.autophagy.lu/ index.html). We used a univariate Cox regression model to calculate the risk ratio (HR) of autophagy-related genes. Then, based on the expression of autophagy-related genes with prognostic effect in 866 ovarian cancer samples in the GEO database, unsupervised cluster analysis was used to identify different autophagy regulation patterns, and patients were classified for further analysis. We used the consensus clustering algorithm of the ConsuClusterPlus package to determine the number and stability of clusters and repeated it 1000 times to ensure the stability of classification [18].

### Gene set variation analysis (GSVA)

We used the "GSVA" R package to analyze the biological process between autophagy regulation patterns [19]. The gene sets "c2. cp.kegg. v7.1" and "h.all.v7.2" were downloaded from MSigDB database, and adjust *p*-value < 0.05 indicates significant significance. The “clusterProfiler “ R package was used to annotate the functions of autophagy-related genes with prognostic values.

### Evaluate the level of immune cell infiltration

We used the CIBERPORT method to quantify the relative abundance of invasion of each immune cell type in ovarian cancer samples. The input matrix is our gene expression matrix, which marks the gene set of each TME infiltrating immune cell type, which is derived from Zhang's study, including innate immune cells and acquired immune cells [20].

### Identification of differentially expressed genes(DEGs) between autophagy regulation patterns

We used the limma R package to analyze DEGs between different autophagy regulation patterns, and *P*-value < 0.05 was regarded as a significant difference. We performed univariate Cox analysis on DEGs, and selected DEGs with survival effects for subsequent analysis.

### Construct the Prognostic Signature for ovarian cancer patients

After multivariate Cox regression analysis of the above DEGs, the risk score of each patient was established. The risk score was calculated as follows: Risk score = ${\sum }_{i=1}^{n}{Coef}_{i }*{x}_{i}$, Where Coef is the coefficient and x is the expression value of each gene. Ovarian cancer patients were divided into high-risk groups and low-risk groups according to the median risk score. Then, we used the survival curve to evaluate the survival difference between the two groups, and the ROC curve to predict the diagnostic value of the prognostic signature (with R package “time ROC”).

### Chemotherapeutic response prediction by Prognostic signature

We analyzed the response of each ovarian cancer sample to chemotherapeutic drugs based on the pharmacometrics database (the Genomics of Drug Sensitivity in Cancer (GDSC),https://www.cancerrxgene.org/). Using the pRRophetic algorithm, a ridge regression model was constructed according to the expression profile of the GDSC cell line and our mixed gene expression profile to predict drug IC50. The parameter settings are as follows: Use "combat" to remove the batch effect and tissue type of "allSoldTumours,” and other parameters are the default values [21].

### Statistical analysis

All statistical analyses were performed with R software (R 4.0.4). Wilcoxon test was used to compare the differences between the two samples, the log-rank test was used to test the survival curve, and the univariate Cox regression model was used to calculate the risk ratio (HR). Pearson method was used to calculate the correlation coefficient of autophagy-related genes, and the 'survivalroc' R package was used to analyze the ROC curve. In addition, *p*-value < 0.05 was considered significant.

## Results

### Consensus cluster analysis was performed by autophagy-related genes

**Fig. 1:**
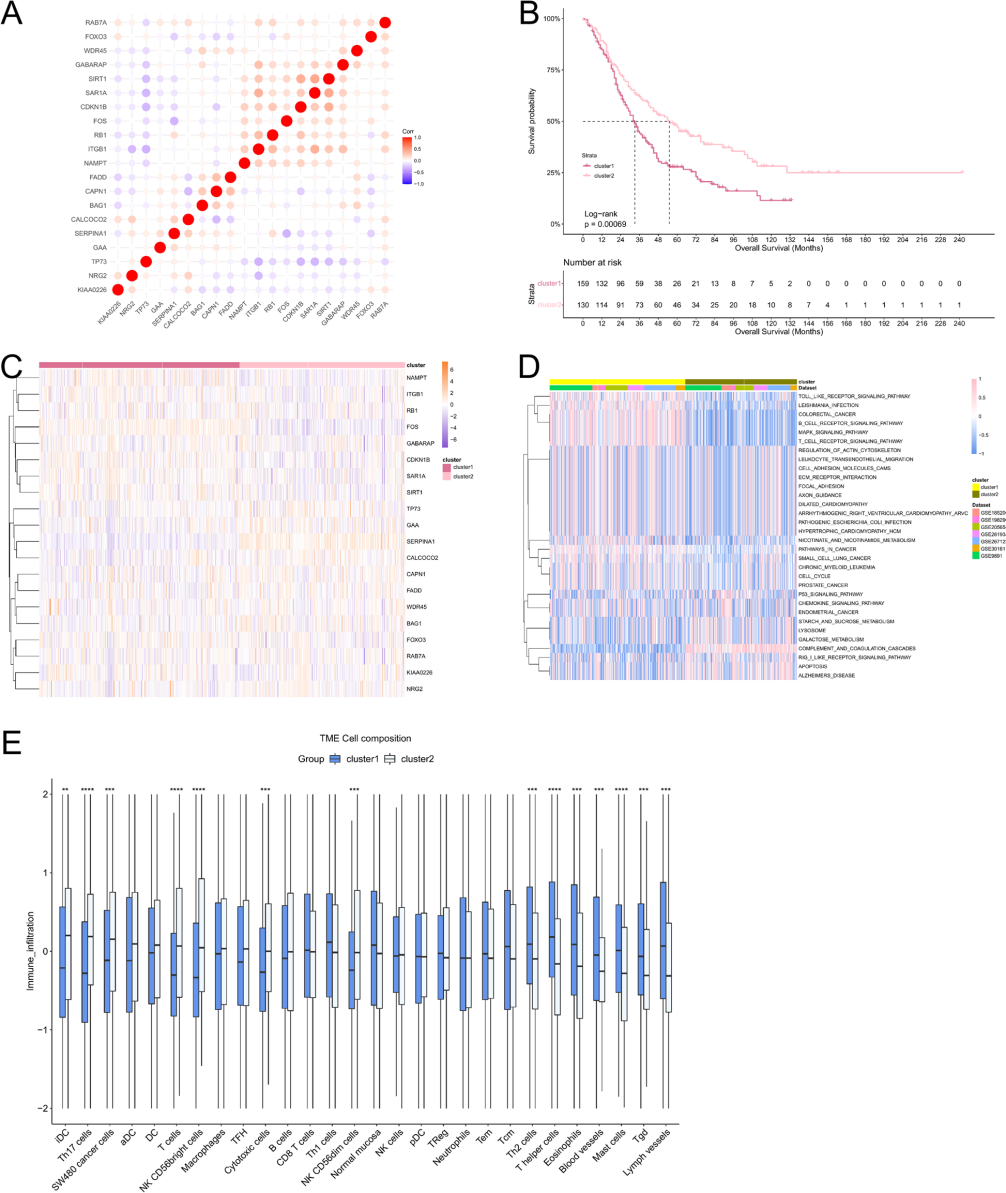
Biological functions of each autophagy regulation pattern. **A** Heatmap shows the positive(red) and negative(blue) correlation between autophagy-related genes in ovarian cancer. **B** The survival curve compared the survival differences between the two autophagy regulation patterns. **C** A heatmap shows the expression of autophagy-related genes between autophagy regulation patterns. **D** GSVA enrichment analysis shows the activation status of biological pathways in different autophagy regulation patterns. Red represents the activation pathway and blue represents the inhibition pathway. **E** CIBERSORT algorithm calculates the relative abundance of TME immune cell infiltration in different autophagy regulation patterns

Seven GEO datasets were selected for analysis (GSE9891, GSE18520, GSE19829, GSE20565, GSE26193, GSE30161, GSE26712) (Additional Table 1). 232 autophagy-related genes were analyzed by univariate cox analysis (Additional Table 2), and 20 autophagy genes had prognostic values (Additional Table 3 and Additional Fig. 1). The above autophagy-related genes were selected for follow-up analysis. To explore the correlation between autophagy-related genes, we made a Pearson correlation of the expression of 20 autophagy-related genes and found that the proportion of positive correlation was greater than negative correlation (Fig. 1A), suggesting that autophagy-related genes may regulate biological function through synergy.

Next, we performed consensus clustering to classify samples from seven GEO datasets based on the expression profiles of 20 autophagy-related genes. After unsupervised clustering, the samples are divided into three categories, of which 306 samples in cluster1, 253 samples in cluster2, and 307 samples in cluster3. After prognostic analysis of the three autophagy regulation patterns, it could be found that the prognosis of the three types of regulation patterns was significantly different, of which cluster2 has the best prognosis and cluster1 had the worst prognosis (Additional Fig. 2). To better analyze the differences between regulation patterns, we selected cluster1 and cluster2 with the most significant difference in survival for follow-up analysis (Fig. 1B, and C).

**Fig. 2:**
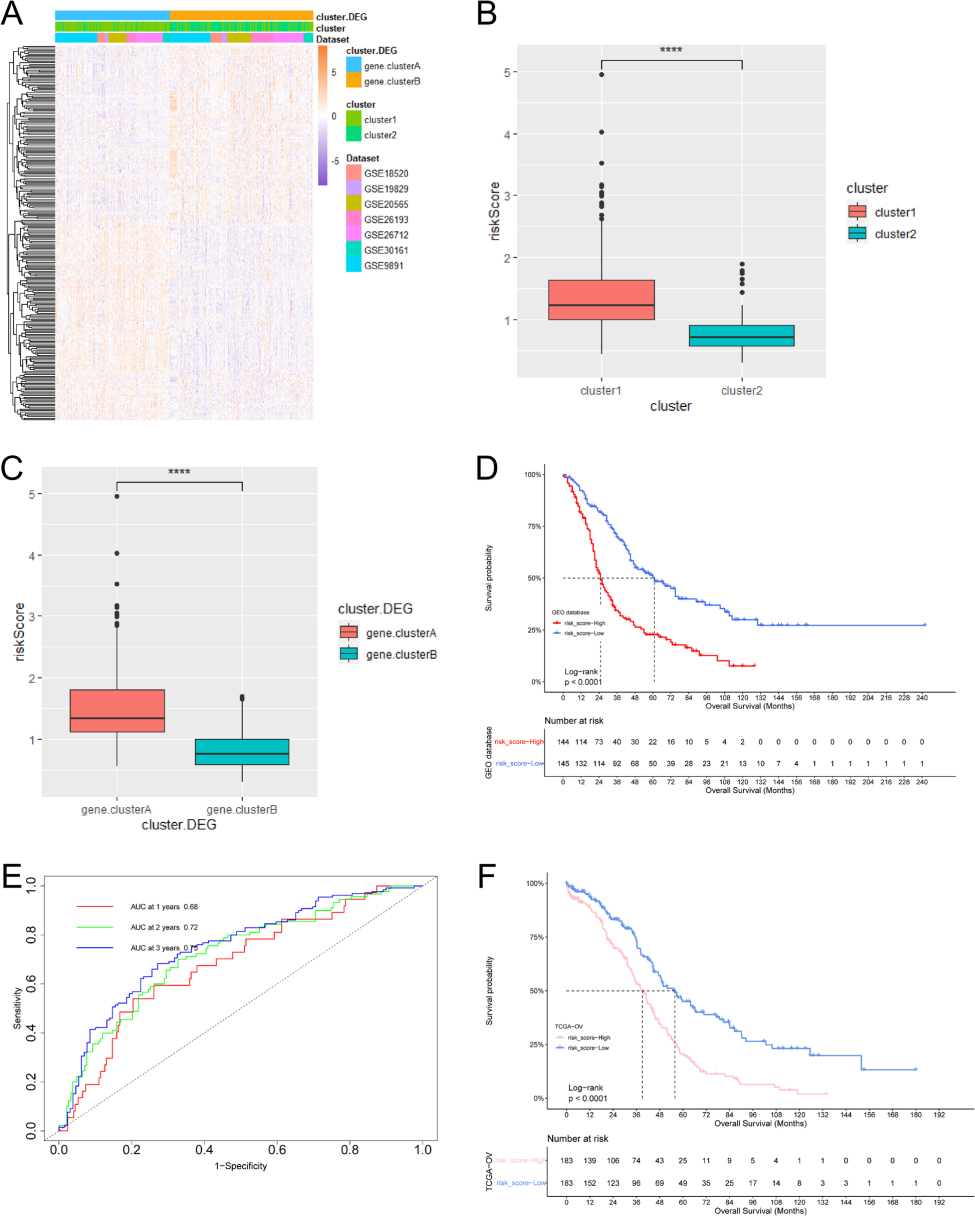
Construction of autophagy-related risk model. **A** Heatmap shows gene expression between different autophagy phenotype regulation patterns. red: high expression; blue, low expression. **B** and **C** The difference of risk score between autophagy regulation patterns (**B**) and autophagy phenotypic regulation patterns (**C**) (t.test, *P* < 0.0001). **D** and **F** The survival curve shows the prognostic difference between high and low-risk groups in GEO database (**D**) and TCGA database (**F**), and the predictive value of the risk model for ovarian cancer patients in GEO database (**E**)

To determine the biological significance of different regulatory patterns, we performed a GSVA enrichment analysis. Cluster1 significantly enriched in carcinogenic related signal pathways, such as ECM receptor interaction, cell adhesion, and MAPK signaling pathways. The signal pathways of cluster2 enrichment include lysosome-related pathway, p53 apoptosis pathway, coagulation, and complement cascade reaction signal pathway (Fig. 1D). Through GSVA enrichment analysis, we found that there may be a certain correlation between immune microenvironment and autophagy regulation patterns. We used the CIBERPORT method to component differences of immune cell infiltration between different patterns. The results showed that innate immune cells [natural killer (NK) cells, NK CD56^dim^cells, NK CD56 ^bright^ cells, plasmacytoid dendritic cells (DCs), immature DCs, activated DCs, and macrophages] were mainly enriched in cluster 2, while adaptive immune cells [Th2 cells, T helper cells] were enriched in cluster 1 (Fig. 1E).

### Construction of autophagy-related risk model

To better study the function of autophagy regulation mode, we extracted 250 autophagy phenotype-related difference genes with limma R package and enriched DEGs with GO and KEGG. We found that DEGs mainly showed cell proliferation in biological processes, and they have also enriched in TNF signaling pathway, MAPK signaling pathway, and PI3K-Akt signaling pathway (Additional Fig. 3 and 4).

**Fig. 3:**
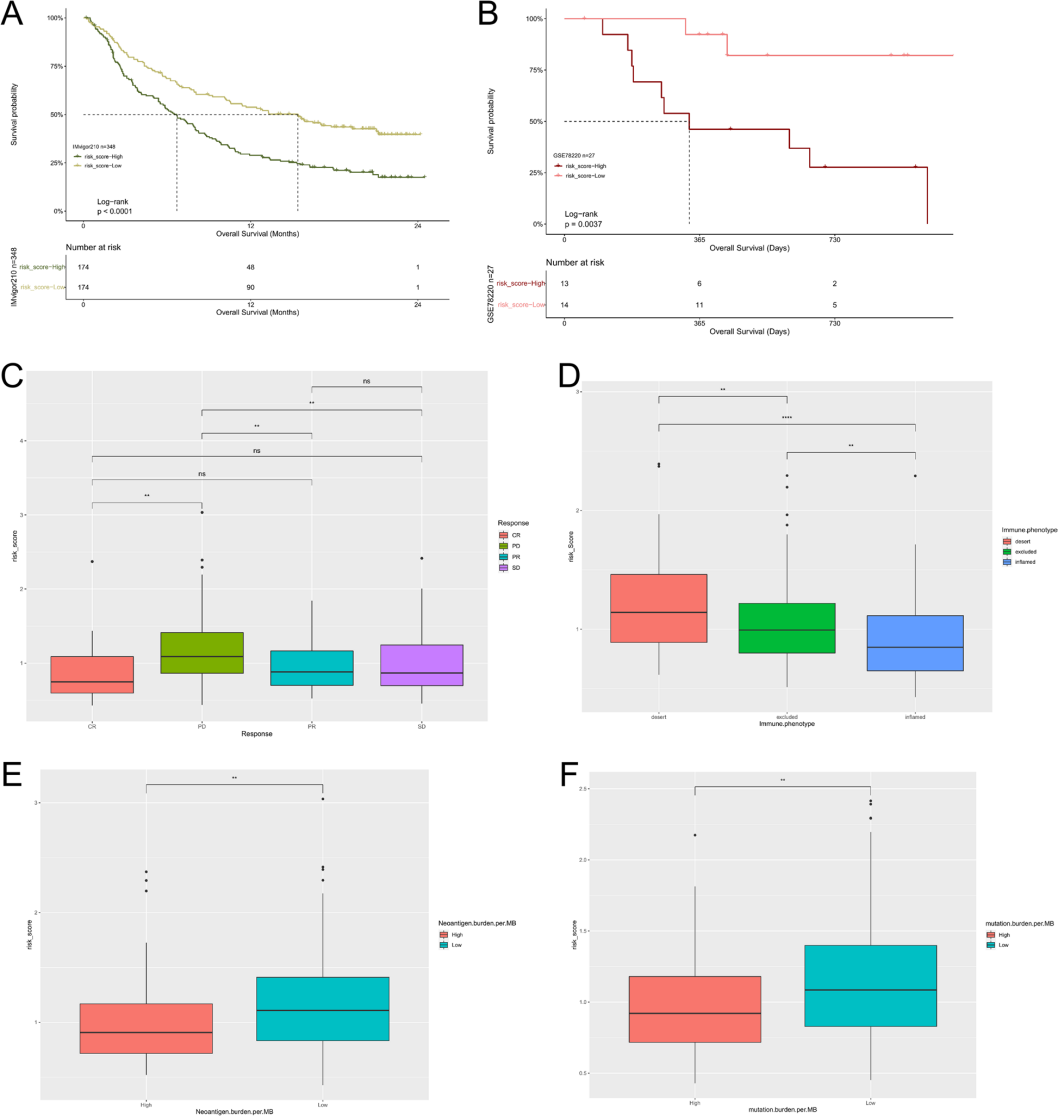
Relationship between risk model and efficacy of immunotherapy. **A** and **B** The survival curve showed the survival difference between the high-risk score and low-risk score groups in the immunotherapy imvigor210 cohort (**A**) and GSE78220 cohort (**B**). **C** In the imvigor210 cohort, there were differences in risk scores between different clinical responses to anti-PD-L1 treatment. **D** Differences in risk scores for different immunophenotypes in the imvigor210 cohort. **E** and **F** There were differences in risk scores between different neoantigen burdens (**E**) and mutation burden (**F**) in the imvigor210 cohort

**Fig. 4:**
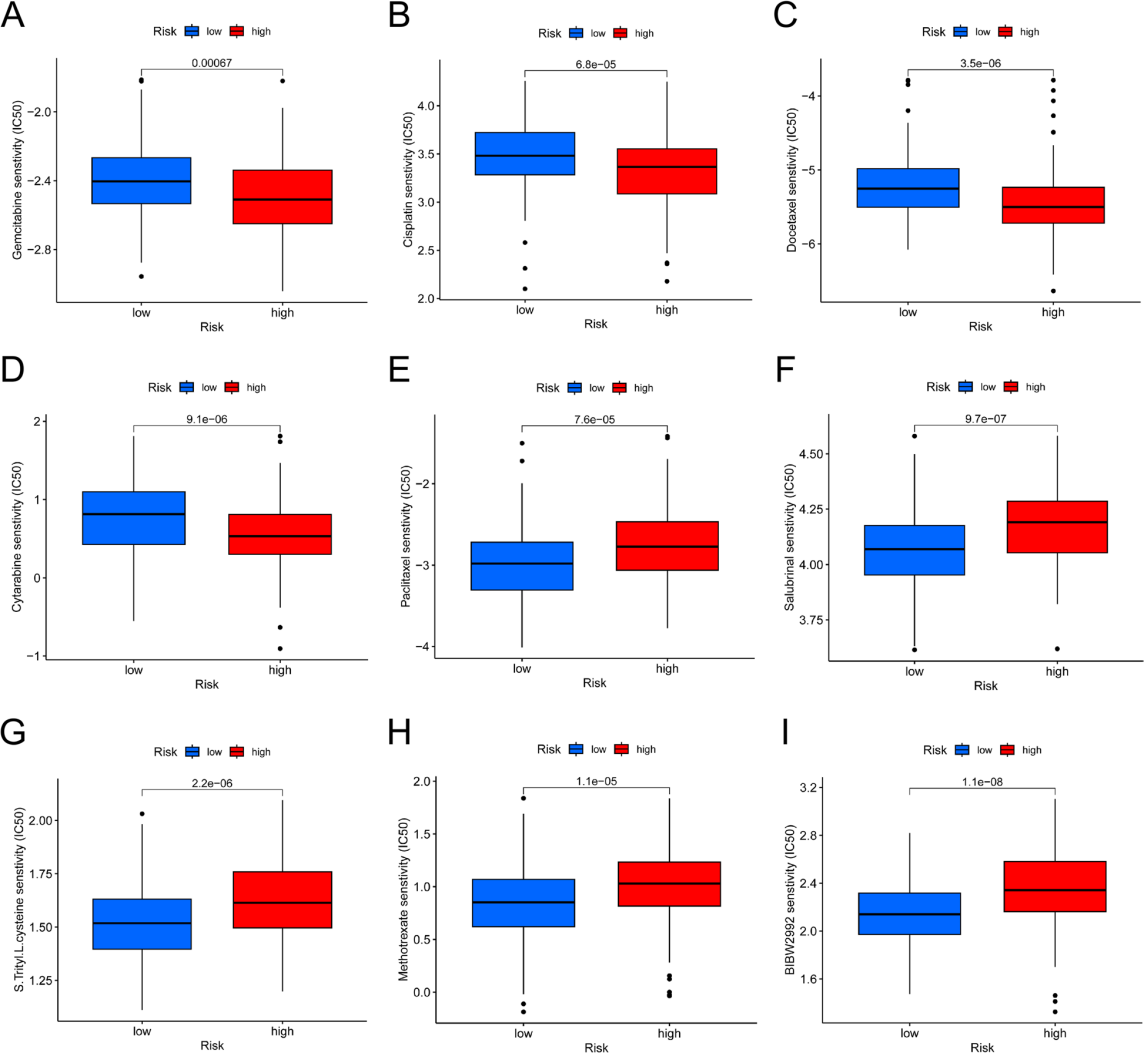
Prediction of drug sensitivity (**A–D**) Sensitive drugs in high-risk score groups. **E-I** Sensitive drugs in low-risk score groups

To further understand this regulatory difference, we performed unsupervised cluster analysis on 250 differential genes. This analysis divided patients into two categories: gene.clusterA and gene.clusterB. Gene.clusterA contained 248 samples and gene.clusterB contained 311 samples. To our surprise, the sample distribution of gene.clusterA was highly consistent with cluster1, and gene.clusterB was highly consistent with cluster2 (Fig. 2A). Survival analysis showed that gene.clusterA had a worse prognosis than gene.clusterB which was similar to the prognostic trend between cluster1 and cluster2 (Additional Fig. 5).

By univariate and multivariate COX regression analysis of 250 DEGs, we obtained six genes that independently predicted survival (Additional Fig. 6). To better quantify the autophagy regulation patterns of ovarian cancer patients, we constructed a risk model based on these six genes. We found that the risk score of cluster1 was significantly higher than that of cluster2, and the risk score of gene.clusterA was also higher than that of gene.clusterB (Fig. 2B, and C). To evaluate the impact of the risk model on the immune microenvironment, we compared immune cell infiltration in different risk score groups (Additional Table 4). We found that Th2 cells, Tgd cells, eosinophils cells, and lymph vessels cells were higher infiltration in the high-risk score group, and aDC cells, NK cd56^dim^cells, and NK CD56^bright^ cells were higher infiltration in the low-risk score group (Additional Fig. 7). To judge the clinical predictive value of the risk model for ovarian cancer patients, we divided the patients into high-risk groups and low-risk groups according to the median value of the risk score. The survival analysis of the two groups showed that the low-risk group had a better prognosis (Fig. 2D). The ROC curve showed that the areas under the curve of 1-year, 3-year, and 5-year overall survival times were 0.68, 0.72, and 0.75 respectively (Fig. 2E), suggesting that the risk model had high accuracy and sensitivity. 366 ovarian cancer samples from the TCGA also verified the accuracy of the risk model (Fig. 2F). These results show that the risk model based on autophagy phenotype-related genes can accurately predict the prognosis of patients.

### The risk model predicts the response to immunotherapy

At present, immunotherapy had provided important clues for the clinical treatment of cancer. Drugs based on PD-L1 and PD-1 blockers have become the research hotspot of tumor immunotherapy. In the previous results, we found that the risk model had a certain correlation with the immune microenvironment. Therefore, through the analysis of two immunotherapy cohorts, we studied whether the risk model could predict the response of ovarian cancer patients to immune checkpoint blocking therapy. We found that the low-risk score group had a longer survival time in both the anti-PD-L1 cohort (IMvigor210) and anti-PD-1 cohort (GSE78220) (Fig. 3A, and B). In the IMvigor210 cohort, patients had different degrees of efficacy in anti-PD-L1 blocker treatment, and patients with complete response had lower risk scores than patients with other reactions (SD, stable disease; PD, progressive disease; CR, complete response; PR, partial response) (Fig. 3C). We also studied the risk score of three immune subtypes in the IMvigor210 cohort and found that the risk score of the "immune infected" subtype was the lowest, while the risk score of the "immune desert" subtype was the highest (Fig. 3D). In addition, the neoantigen burden and mutation burden in the low-risk score group were significantly higher than those in the high-risk group (Fig. 3E, and F), suggesting that patients in the low-risk group may be more likely to stimulate an immune-inflammatory response.

### Prediction of drug sensitivity

Since chemotherapy is a common treatment for ovarian cancer, we evaluated the response of the risk model to chemotherapy drugs for ovarian cancer. Based on the GDSC drug database, we evaluated the IC50 value of each sample of the GSE mixing matrix for the drug. We could find that the IC50 values of these chemotherapeutic drugs were different between the high-risk score group and the low-risk score group, and the patients with low-risk scores may be more sensitive to paclitaxel, salubrinal, S.Trityl.L.cysteine, Methotrexate and BIBW2992 (Fig. 4E-I), while the high-risk group was more sensitive to docetaxel, cisplatin, Cytarabine and gemcitabine chemotherapy (Fig. 4A-D). Paclitaxel combined with platinum drugs is the first-line treatment of ovarian cancer chemotherapy. The analysis of drug sensitivity can provide the basis for the development of personalized treatment for tumor patients.

## Discussion

Human homeostasis depends on the interaction of a variety of complex regulatory mechanisms. More and more studies have shown that autophagy plays an important role in the occurrence and development of tumors by participating in biological activities such as inflammation, immune response, and oxidative stress [13]. Although immune checkpoint inhibitors (ICB) have not been approved as conventional drugs for the treatment of ovarian cancer, immunotherapy in urothelial cancer and melanoma had achieved good clinical effects [12, 17]. Therefore, we could predict the possibility of immunotherapy for ovarian cancer by analyzing the immune microenvironment. Previous studies on immune microenvironment and autophagy have often focused on a single immune cell type or regulatory molecule [9, 10, 22]. The overall infiltration characteristics of TME mediated by integrating multiple autophagy-related genes have not been fully analyzed. Identifying the role of autophagy regulation mode in the immune microenvironment will help to improve our understanding of TME anti-ovarian cancer immune response, and guide more effective clinical treatment strategies.

In this study, we first obtained 232 reported autophagy-related genes from the Human Autophagy Database, and then selected 20 prognostic autophagy-related genes according to the mixed gene expression profile on the GEO database. Based on 20 prognostic autophagy-related genes, we developed two different autophagy regulation patterns. These two models had significantly different prognostic effects and characteristics of TME cell infiltration. The prognosis of cluster1 was significantly worse than that of cluster2, and adaptive immune cells were mainly enriched in cluster1, while innate immune cells were mainly enriched in cluster2. Through GSVA enrichment of two autophagy regulation patterns, it was found that carcinogenic pathways were mainly enriched in cluster1, including ECM receptor interaction, cell adhesion, and MAPK signaling pathways, while cluster2 was mainly enriched in apoptotic pathway and lysosomal pathway. This may be one of the reasons why cluster 2 had a better prognosis than cluster1. Then, based on two different autophagy regulation patterns, we identified two ovarian cancer subtypes related to autophagy regulation patterns: gene.clusterA and gene.clusterB. To quantify the efficacy of autophagy regulation patterns in individual ovarian cancer patients, we constructed a risk score model based on the two ovarian cancer subtypes. We found that the high-risk score group had a worse prognosis than the low-risk score group. According to the histogram, we analyze the sample overlap of these three different classification patterns and found that the three classification patterns were highly consistent in the sample composition (Additional Figs. 8 and 9), and they were also similar in prognostic differences and types of immune cell infiltration. This shows that our classification model had good stability. We found that in the IMvigor210 cohort and GSE72880 cohort, the prognosis of the high-risk group was worse than that of the low-risk group, and showed a higher risk score in the immune desert phenotype, while the risk score of the immune-inflammatory phenotype was lower. Immune desert phenotype could be regarded as the non-inflammatory tumor, also known as a "cold tumor". Although there are a large number of immune cells, they may not be involved in regulating tumor progression [23, 24]. The immuneinflamed phenotype was called "hot tumor", which means that more immune cells were infiltrating the tumor microenvironment [25–27]. In the IMvigor210 cohort, we also found that among patients with complete response to PD-L1 blockers, the risk score was the lowest. Previous studies had shown that a high neoantigen burden and high mutation load can increase the lasting clinical benefits of immunotherapy [28]. In our model, the risk scores of the high mutation load group and high neoantigen burden group were lower, which was consistent with the results of previous studies, which indicates that the risk model we constructed could predict the patient's response to ICB treatment. Finally, we used the GDSC database to analyze the therapeutic guidance role of the risk model in ovarian cancer. We found that the low-risk group was more sensitive to paclitaxel, salubrinal, S.Trityl.L.cysteine, Methotrexate and BIBW2992, while the high-risk group was more sensitive to docetaxel, cisplatin, Cytarabine and gemcitabine chemotherapy. In our team's previous studies, we found that autophagy can reverse cisplatin resistance in ovarian cancer [29–31], which coincides with the conclusion that there was a significant difference in sensitivity to cisplatin between high-risk groups, which may provide preliminary evidence for future treatment of ovarian cancer targeting autophagy. By analyzing the immune cell infiltration of different autophagy regulation patterns identifying the risk scores of different tumor immunophenotypes, and judging the sensitivity of ovarian cancer patients to conventional chemotherapy, we provide a new direction for formulating personalized anti-cancer treatment.

## Conclusions

In general, we used the ovarian cancer cohort of TCGA and GEO database to determine a risk model based on autophagy-related genes, systematically and comprehensively analyzed the risk models, revealed their impact on the prognosis of ovarian cancer patients, and provided some ideas for patients' immunotherapy by analyzing the immune microenvironment. Our findings suggest that risk models based on autophagy-related genes may help to promote personalized medicine in the clinical environment.

## Supplementary Information

**Additional file 1:** **Supplementary Table 1.** Eight GSE datasets for ovarian cancer cohorts. **Table 2.** 232 autophagy related genes. **Table 3.** 20 autophagy related genes with prognostic value. **Table  4.** 28 types of immune cells.

**Additional file 2:** **Supplementary Figure 1.** 20 autophagy related genes with prognostic value after univariate COX regression analysis. **Figure 2.** Survival curves of three types of autophagy regulation patterns. **Figure 3** and **4.** GO(S2) and KEGG(S3) enrichment of differentially expressed genes (DEGs). **Figure 5.** Survival curves of two autophagy phenotype regulation patterns. **Figure 6.** Six autophagy phenotype related genes with independent prognostic value after multivariate COX analysis. **Figure 7.** Differences of immune cell infiltration between high and low- risk score groups. **Figures 8** and **9.** Sample composition of three classification models.

## Data Availability

The direct links required to find each dataset in the database are as follows: the GEO gene expression and clinical dataset: https://www.ncbi.nlm.nih.gov/geo/; the TCGA gene expression and clinical dataset: https://portal.gdc.cancer.gov/repository. IMvigor210 cohort: http://research-pub.gene.com/IMvigor210CoreBiologies.The dataset downloaded by this direct link is the original dataset.

## Abbreviations

ICB: Immune checkpoint inhibitor
PRR: Pattern recognition receptor
TME: Tumour Microenvironment
DEGs: Differentially expressed genes
OV: Ovarian cancer
HADb: Human Autophagy Database
GDSC: The Genomics of Drug Sensitivity in Cancer
GO: Gene oncology
KEGG: Kyoto encyclopedia of genes and genomes
SD: Stable disease
PD: Progressive disease
CR: Complete response
PR: Partial response
ROC: Receiver operator characteristic curve
