# Supplementary material for: Development of a risk model based on autophagy-related genes to predict survival and immunotherapy response in ovarian cancer
Source: Hereditas. 2023 Feb 1;160:4. doi: 10.1186/s41065-023-00263-2 (PMC9890868; doi:10.1186/s41065-023-00263-2)
Supplement: Supplementary file 2 — Additional file 2: Supplementary Figure 1. 20 autophagy related genes with prognostic value after univariate COX regression analysis. Figure 2. Survival curves of three types of autophagy regulation patterns. Figure 3 and 4. GO(S2) and KEGG(S3) enrichment of differentially expressed genes (DEGs). Figure 5. Survival curves of two autophagy phenotype regulation patterns. Figure 6. Six autophagy phenotype related genes with independent prognostic value after multivariate COX analysis. Figure 7. Differences of immune cell infiltration between high and low- risk score groups. Figures 8 and 9. Sample composition of three classification models. [file 41065_2023_263_MOESM2_ESM.docx]

**Additional Figures**


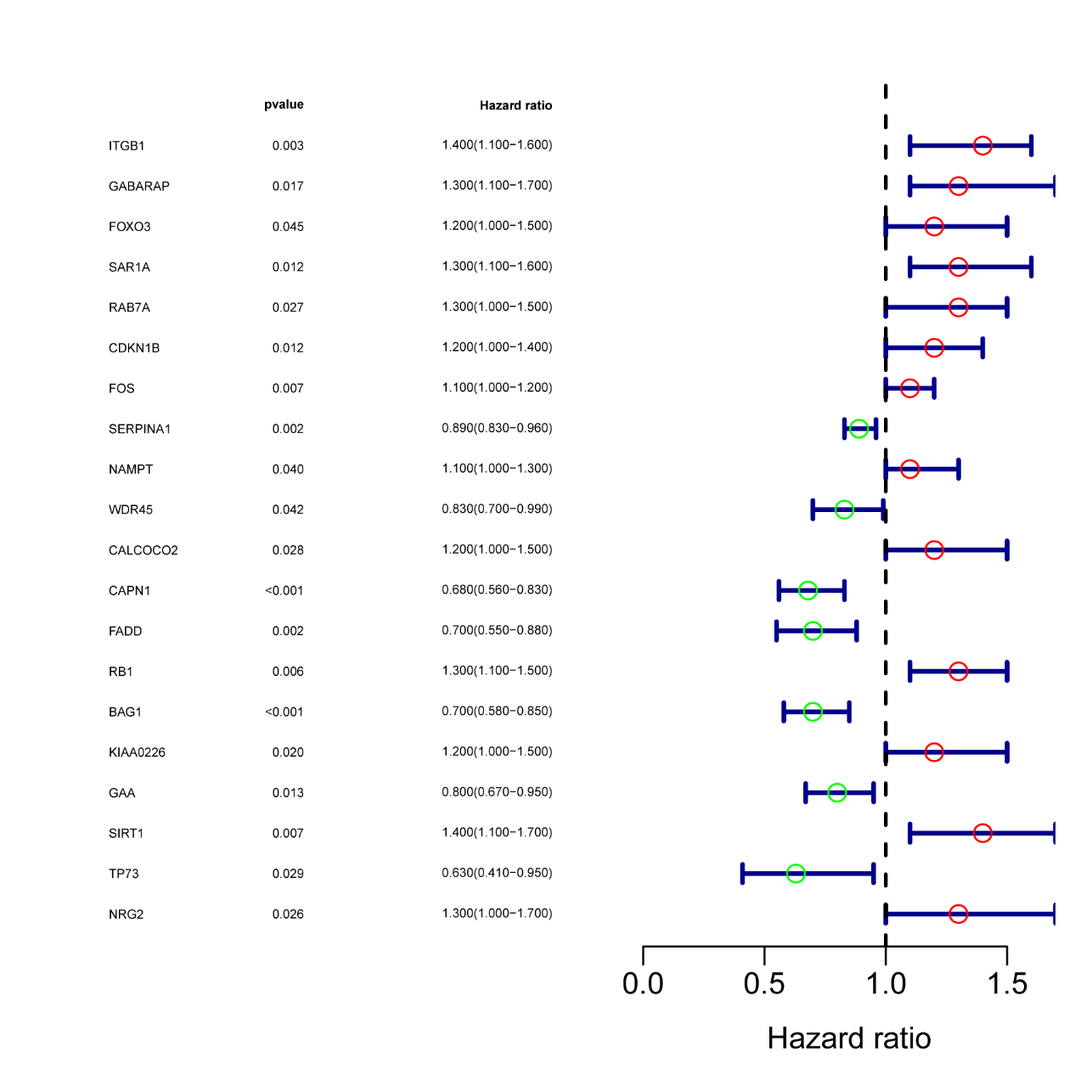
 **Figure**1 20 autophagy related genes with prognostic value after univariate COX regression analysis. (tif).


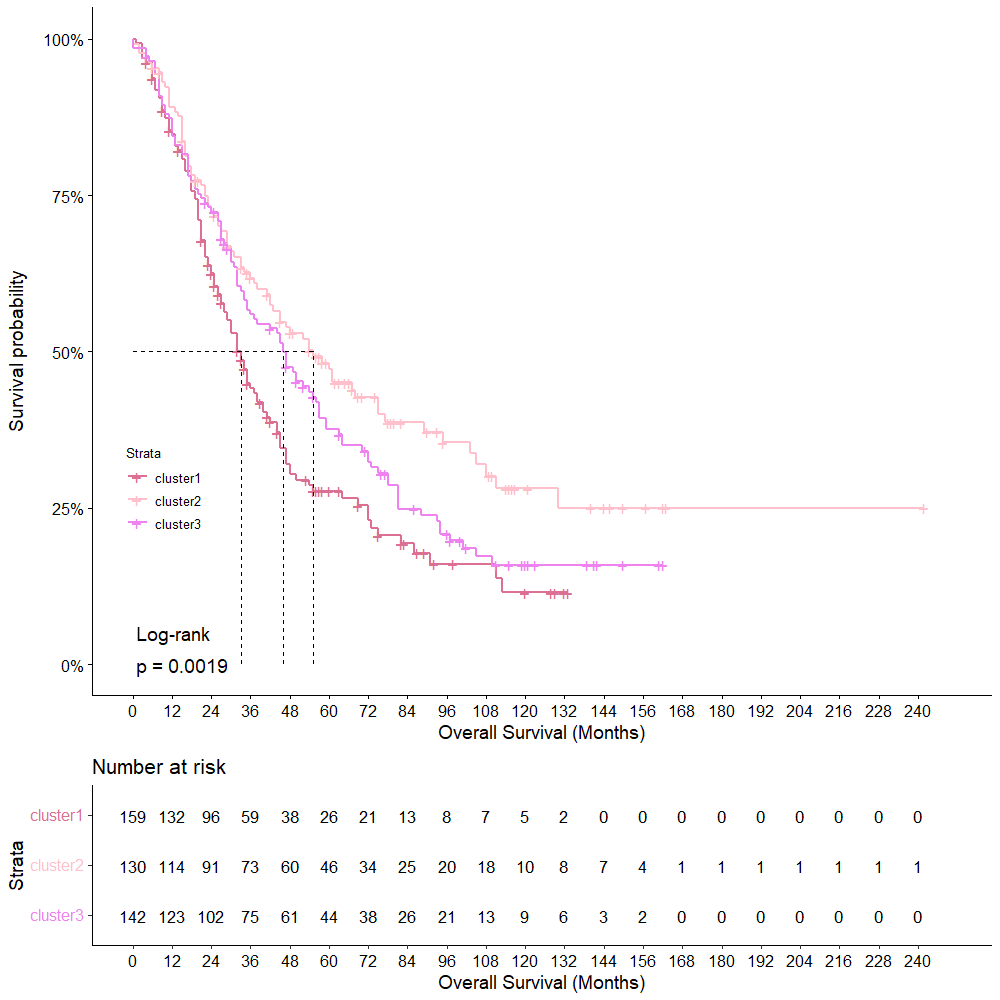


**Figure 2**. Survival curves of three types of autophagy regulation patterns. (tif).


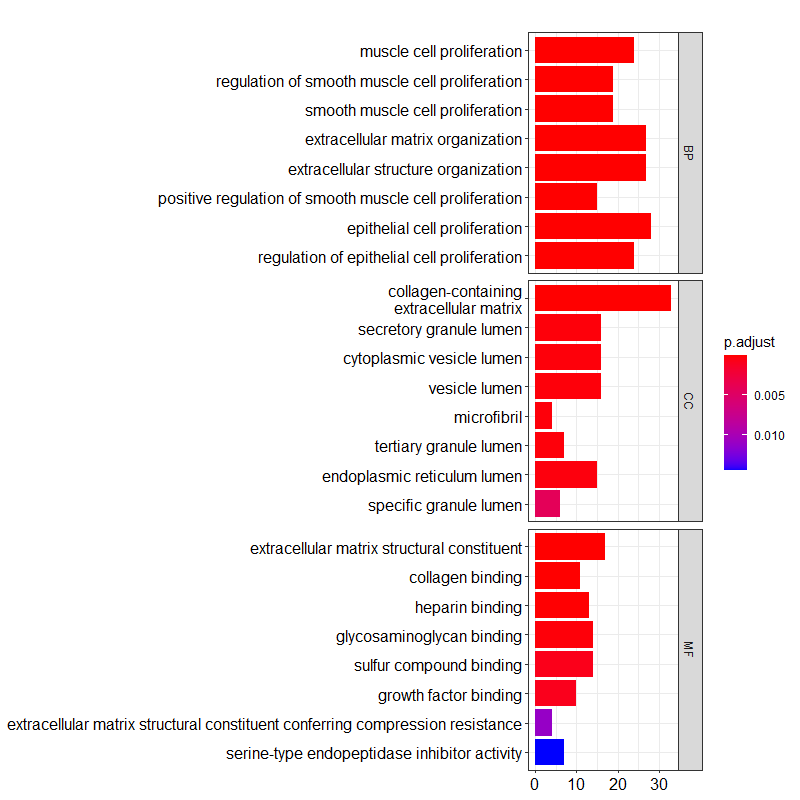


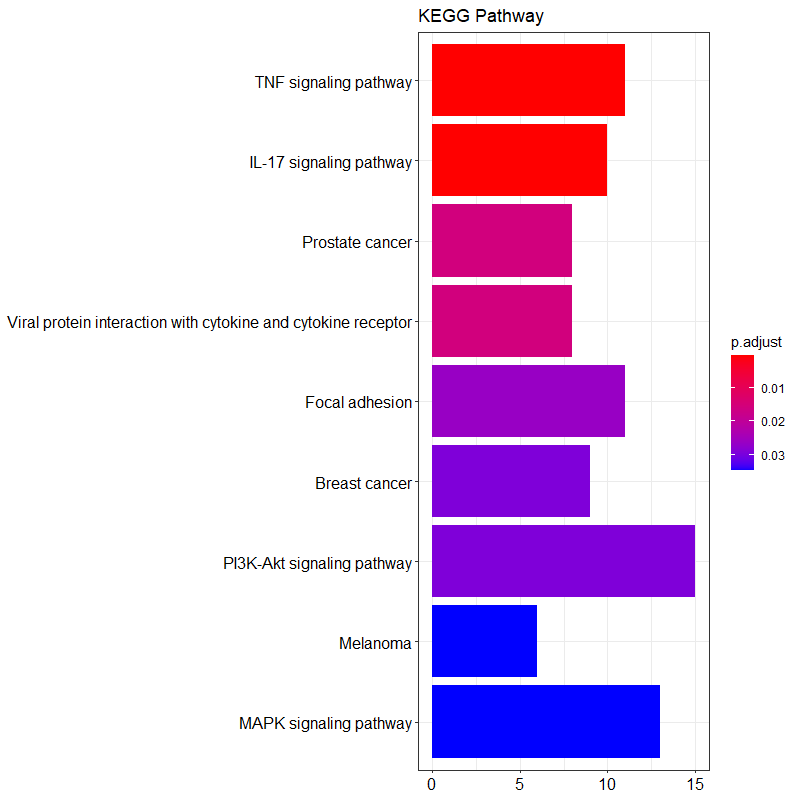


**Figure 3 and 4.** GO(S2) and KEGG(S3) enrichment of differentially expressed genes (DEGs). (tif)**.**


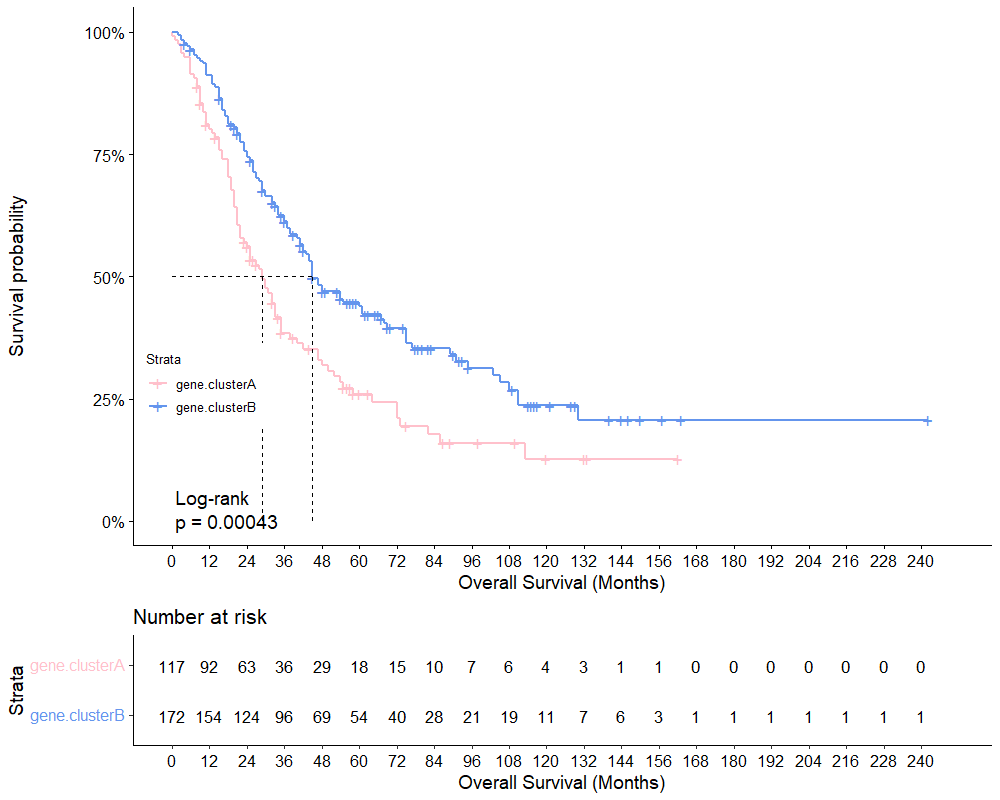


**Figure 5.** Survival curves of two autophagy phenotype regulation patterns. (tif).


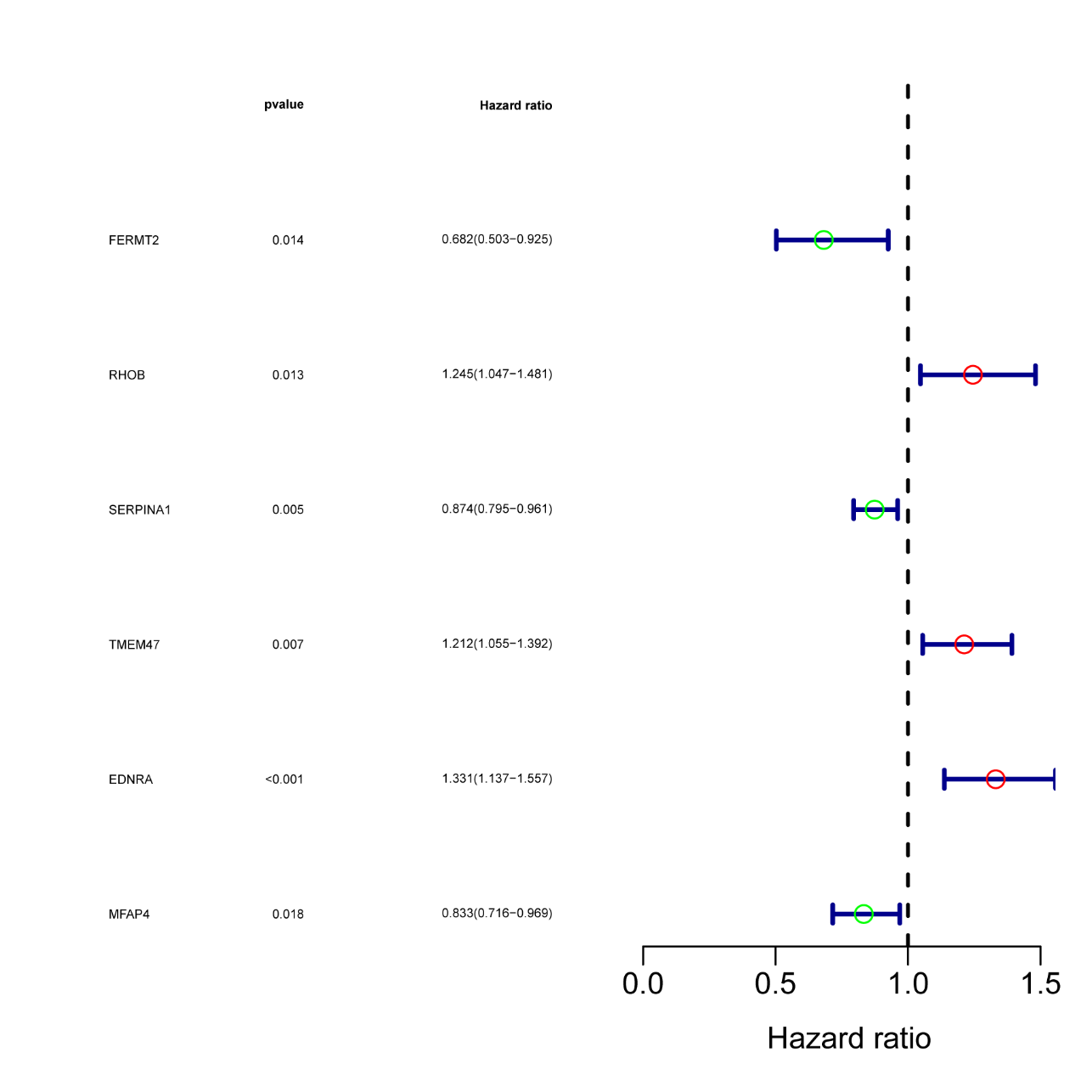
 **Figure 6.** Six autophagy phenotype related genes with independent prognostic value after multivariate COX analysis. (tif).


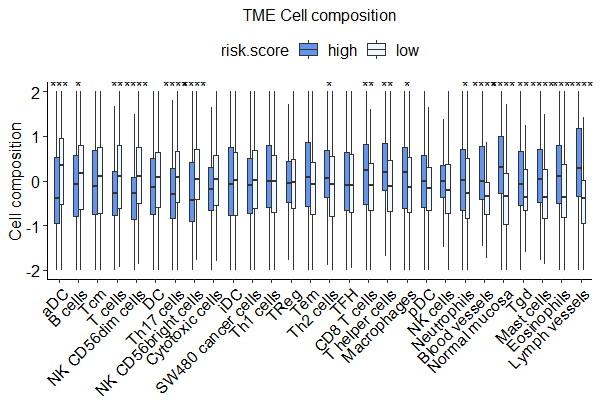
 **Figure 7.** Differences of immune cell infiltration between high and low- risk score groups. (tif)**.**


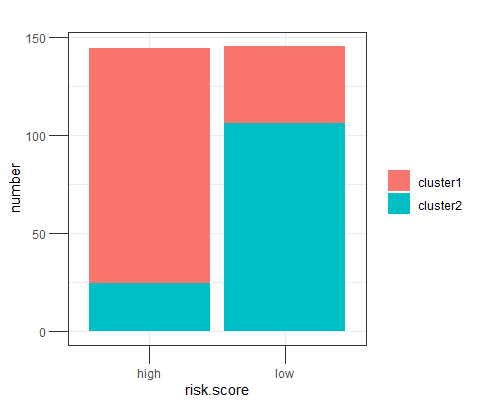

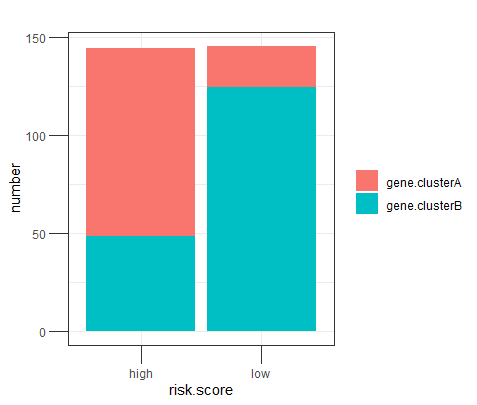


**Figure 8 and 9.** Sample composition of three classification models**.** (tif)
